# Prostatites aiguës sur prostate non tumorale aux cliniques universitaires de Lubumbashi: aspects épidémio-clinique et thérapeutique

**DOI:** 10.11604/pamj.2020.37.290.21260

**Published:** 2020-12-01

**Authors:** Manix Ilunga Banza, Trésor Kibangula Kasanga, Augustin Kibonge Mukakala, Yannick Tietie Ben N'dwala, Christelle Ngoie Ngoie, Vincent De Paul Kaoma Cabala, Néron Tapenge Shutsha, Lire Ipani Lire, Eric Wakunga Unen, Nathalie Dinganga Kapessa

**Affiliations:** 1 Département de Chirurgie, Faculté de Médecine, Cliniques Universitaires de Lubumbashi, Université de Lubumbashi, Province du Haut Katanga, Ville de Lubumbashi, République Démocratique du Congo,; 2 Département de Chirurgie, Faculté de Médecine, Cliniques Universitaires de Bukavu, Université Officielle de Bukavu, Bukavu, République Démocratique du Congo

**Keywords:** Prostatites, aiguës, non tumoral, épidémiologie, Prostatitis, acute, noncancerous, epidemiology

## Abstract

**Introduction:**

les prostatites aiguës sont une entité fréquente en urologie. L'objectif de cette étude était d'analyser les aspects épidémio-cliniques et thérapeutiques des prostatites aiguës sur des prostates non tumorales aux Cliniques Universitaires de Lubumbashi.

**Méthodes:**

il s'est agi d'une étude descriptive transversale et rétrospective rapportant une série de 25 patients souffrant de prostatite aiguë documentée et pris en charge aux Cliniques Universitaires de Lubumbashi durant une période de quatre ans soit de 2015 à 2018. Tous les patients porteurs de tumeurs prostatiques ont été exclus de notre étude. Les données ont été recueillies sur base d´une fiche d´enquête reprenant différents paramètres d´étude répartis en 3 catégories à savoir les données épidémiologiques comprenant l´âge, la période d´étude, la résidence, les données cliniques reprenant les signes subjectifs, les signes objectifs, l´état général, les éléments du toucher rectal ainsi que les données paracliniques réparties en laboratoires et imagerie.

**Résultats:**

la prostatite aiguë sur prostate non tumorale a représenté 1,27% de l´ensemble de la pathologie chirurgicale et 7,66% en urologie. La tranche d´âge la plus touchée était celle de 19 à 37 ans avec 64% des cas, l´âge moyen est de 33,16±2,4 ans. Dix-sept patients (68%) étaient suivis en ambulatoires et 8 (32%) en hospitalisation. Sur le plan clinique, la fièvre au-delà de 38,5°celsius était retrouvée chez 15 patients (60%), la dysurie chez 11 patients (44%), rétention aiguë d´urine chez 3 patients (12%), les brulures mictionnelles chez 8 patients (32%), syndrome douloureux chez 21 patients (84%), la sensibilité prostatique au toucher rectal chez 18 patients (72%). Sur le plan de l´imagerie, l´échographie a été le seul examen réalisé et ce, chez 16 patients (64%). Sur le plan biologique, le bilan inflammatoire était quasi-systématique chez tous nos patients (100%) comprenant la NFS, la VS, la CRP; l´hémoculture réalisée chez 4 patients (16%) parmi lesquels 3 étaient positives. Tous nos patients avaient réalisé l´examen cytobactériologique des urines ou des sécrétions prostatiques recueillies par un massage prostatique. La culture d´urine était stérile chez 6 patients (24%) et positive chez 19 patients (76%) avec Escherichia coli comme germe le plus retrouvé, chez 16 patients sur les 19 (84,21%). Tous nos patients ont été mis sous anti-inflammatoire en intra-rectale et les fluoroquinolones ont été les antibiotiques les plus utilisés dans notre série chez 18 patients (64%) parmi lesquels 12 en monothérapie. Six cas sur les 25 (24%) étaient associés à une orchi-épidydimite. La durée de traitement allait de 2 semaines à 4 semaines avec comme critère d´arrêt de traitement soit la stérilisation des sécrétions ou des urines soit la disparition de la leucocyturie. Ainsi, sur les 19 patients avec culture positive à l´admission, 14 ont réalisé une deuxième culture (73,68%) à 2 semaines de traitement parmi lesquels 3 (12%) étaient encore positives et ont dû réaliser une troisième culture 4 semaines après le début de traitement. L´évolution était bonne chez 22 patients (88%) avec une rémission complète des signes cliniques et biologiques et 3 cas (12%) ont évolué vers une persistance des signes et un passage à la chronicité, aucun cas d´évolution vers un abcès prostatique.

**Conclusion:**

la prostatite aiguë sur prostate non tumorale reste une entité nosologique urologique très préoccupante dont la prise en charge doit être rigoureuse d´autant plus que la population à risque est celle en période d´intense activité sexuelle. L´usage de l´échographie endorectale, la proscription du massage prostatique doivent s´intégrer dans la prise en charge aux cliniques universitaires de Lubumbashi.

## Abstract (en)

**Introduction:**

acute prostatitis is a common urological condition. The purpose of this study was to analyze the epidemioclinical features and therapy of acute prostatitis associated with noncancerous prostate at the Lubumbashi University Clinics.

**Methods:**

we conducted a descriptive cross-sectional and retrospective study of a series of 25 patients with documented acute prostatitis and treated at the Lubumbashi University Clinics over a period of four years, from 2015 to 2018. All patients with prostate cancer were excluded from our study. Data were collected via a survey form based on different study parameters divided into 3 categories, namely epidemiological data including age, study period, residence, clinical data with subjective signs, objective signs, general status, findings on rectal examination as well as paramedical data divided into laboratory and imaging tests.

**Results:**

acute prostatitis associated with noncancerous prostate accounted for 1.27% of all surgical diseases and 7.66% in urology. The most affected age group was 19-37 years (64% of cases), mean age was 33.16±2.4 years. Seventeen patients (68%) were followed up in outpatient clinics and 8 (32%) in hospital. Clinically, fever above 38.5°C was found in 15 patients (60%), dysuria in 11 patients (44%), acute urinary retention in 3 patients (12%), burning during urination in 8 patients (32%), pain syndrome in 21 patients (84%), tender prostate on rectal examination in 18 patients (72%). Ultrasound was the only examination performed in 16 patients (64%). Biologically, assessment of inflammation was performed almost systematically in all patients (100%) including complete blood count (CBC), sedimentation rate (SR), C reactive protein (CRP) levels; blood culture was performed in 4 patients (16%), three of whom had positive blood culture. All patients underwent cytobacteriological examination of the urine or prostatic secretions collected by prostate massage. Urine culture was sterile in 6 patients (24%) and positive in 19 patients (76%). Escherichia coli was the most common germ in 16 out of a total of 19 patients (84.21%). All patients received rectal anti-inflammatory drugs. Fluoroquinolones were the most used antibiotics in 18 patients (64%), twelve of whom received antibiotics as monotherapy. Six out of 25 (24%) cases were associated with orchiepididymitis. The lenght of treatment ranged from 2 to 4 weeks, with either sterilization in secretions or urine or disappearance of leukocyturia as the criteria for treatment discontinuation. Thus, out of 19 patients with positive culture on admission, 14 underwent a second culture (73.68%) at 2 weeks of treatment, three of whom (12%) still had positive test and had to undergo a third culture 4 weeks after they had started treatment. Patient's course was good in 22 cases (88%) with complete clinical and biological remission; three patients (12%) persisted in symptoms which became chronic; no patients had prostatic abscess.

**Conclusion:**

acute prostatitis associated with noncancerous prostate is a really worrying urological, nosologic condition whose management must be rigorous, especially in people at risk, namely those with intense sexual behaviour. Endorectal ultrasound and prostate massage should be integrated into patient care at the Lubumbashi University Clinics.

## Introduction

La prostatite se définit comme étant une inflammation aiguë ou chronique de la glande prostatique, d´origine infectieuse (bactérienne le plus souvent) ou non bactérienne [1,2]. Le terme de prostatite s´emploi chez l´homme adulte pour désigner des plaintes variées localisées dans la région périnéale et le tractus urinaire inférieur. La prévalence des prostatites varie de 8% à 16% dans la population générale [3] et on estime à 50% les hommes qui vont présenter à un moment de leur vie une affection prostatique [4]. Les prostatites sont des affections relativement fréquentes chez les hommes de moins de 50 ans, à l´origine de plus de 2 millions de consultation par an aux USA, où sa prévalence est estimée actuellement entre 5 et 8,8% [5,6]; certaines littératures affirment que c´est un véritable problème de santé publique [4] touchant l´homme du berceau au fauteuil roulant, l´adénome et le cancer étant des affections de l´homme âgé [7].

Aux USA, c´est le diagnostic urologique le plus courant chez l´homme de moins de 50 ans et le troisième chez l´homme de plus de 50 ans après l´hypertrophie bénigne de la prostate et le cancer de la prostate, représentant de 8% [8] à 25% [9] des visites chez l´urologue; c´est une entité extrêmement rare en pédiatrie [10]. La prostatite apparait d’augmenter le risque de développer une hypertrophie bénigne de la prostate et la possibilité de cancer de la prostate [11,12]. La classification traditionnelle distingue les prostatites aiguës associant un syndrome infectieux général, accompagné d´une altération de l´état général, des troubles mictionnels et surtout, d´une prostate douloureuse au toucher rectal, et les prostatites chroniques regroupant toutes les autres pathologies associant des troubles mictionnels, des douleurs pelviennes ou périnéales ainsi qu´un éventuel écoulement urétral [13].

La classification de la *National Institute of Health* est maintenant communément admise par la communauté urologique internationale [14,15] et tous les cliniciens; basée sur les signes présentés par le patient et la présence ou non des leucocytes dans les sécrétions prostatiques ou dans le sperme. Cette classification plus objective, basée sur des critères histologiques et bactériologiques, reposant sur l´analyse des urines et du liquide prostatique; elle permet le calcul d´un index (chronic prostatitis symptom index (CPSI)) autorisant la quantification des symptômes et l´étude de l´efficacité des traitements, mais des véritables études cliniques sont quasi-inexistantes et aucune méta-analyse n´a été réalisée [16,17]. Elle permet d´individualiser quatre catégories (Tableau 1): les prostatites bactériennes aiguës (catégorie 1), les prostatites bactériennes chroniques (catégorie 2), les prostatites chroniques non bactériennes (catégorie 3) et les prostatodynies (catégorie 4).

**Tableau 1:** classification des prostatites

Prostatite bactérienne aiguë (PBA)
Prostatite bactérienne chronique (PBC)
**Syndrome douloureux pelviens chroniques (SDPC)**
SDPC inflammatoire: leucocytes dans le liquide séminal/SPM/VB3
SDPC inflammatoire: leucocytes absents dans le liquide séminal/SPM/VB3
Prostatite inflammatoire asymptomatique (prostatite histologique)

Les prostatites apparaissent comme un groupe très hétérogène. Le diagnostic des prostatites aiguës a toujours été bien individualisé, alors que celui des prostatites chroniques connu depuis le début du XIX^e^ siècle a toujours été beaucoup plus difficile, puis qu´il a fallu attendre Félix Guyon pour distinguer l´adénome prostatique de la prostatite chronique [13]. Les prostatites aiguës sont peu fréquentes que les chroniques (5% de l´ensemble des prostatites) [18] et touchent principalement l´homme adulte entre 40 et 60 ans, pouvant être simples ou à risque des complications [18]. On distingue 2 entités; les prostatites aiguës associées aux soins (notamment après biopsie de la prostate) et les épisodes aiguës sur prostatites chroniques [18]; un tableau d´infection urinaire fébrile de l´homme doit faire évoquer à priori une prostatite aiguë [19].

La prostatite aiguë est une inflammation aiguë d´origine microbienne de la prostate. Elle affecte environ 1% des hommes au cours de leur vie avec des tableaux allant des simples brulures urinaires à des sepsis sévères. Compte tenu de l´existence de la prostate et de la longueur urétrale, les cystites chez l´homme sont rares et toute infection urinaire fébrile avec des signes mictionnels doit faire évoquer une prostatite bactérienne [19]. Le mécanisme pathogénique précis de la prostatite n´est pas connu, la principale théorie est celle d´un reflux d´urines de l´urètre vers les canicules intra-prostatiques entrainant une infection par voie ascendante et conduisant à une prostatite bactérienne ou chimique [20]. La contamination par voie hématogène est rare; enfin la contamination peut être d´origine iatrogène par inoculation directe microbienne au cours d´un cathétérisme urétral rétrograde ou d´une biopsie prostatique [21].

Dans la prostatite aiguë, Lorsque l´agent pathogène (bactérie, virus, champignon etc.) est détecté par l´organisme via la molécule de reconnaissance d´antigène, le système immunitaire produit une réponse inflammatoire aiguë dans le but d´éradiquer l´agent infectieux [22,23]; cette première ligne de défense est l´immunité innée (ou système immunitaire non spécifique ou immunité naturelle). Après la reconnaissance du germe par l´organisme, et l´activation de l´immunité innée, une réponse inflammatoire aiguë se commence avec la sécrétion des variétés de cytokines (tumor necrosis factor alpha-TNF- α) et l´interleukine (Il-1) et chemokines (Il-8, monocytes), les macrophages protéines inflammatoires 1- α), ce qui stimule le recrutement des cellules inflammatoires au site d´infection prostatique [24,25].

Dans sa forme typique, la prostatite aiguë associe un syndrome infectieux de survenue brutale avec fièvre et frissons à des troubles mictionnels (brulures mictionnelles, pollakiurie, dysurie voire rétention aiguë d´urines, parfois rarement peut exister un épisode hématurique initial ou d´urétrorragie; les urines sont troubles). Une prostate globalement augmentée de volume, douloureuse et tendue au toucher rectal [21], dont la consistance est qualifiée de succulente [13]. En plus du tableau clinique, certains examens complémentaires sont nécessaires pour le diagnostic, pour rechercher une complication, planifier l´attitude thérapeutique et suivre l´évolution [4].

L´identification bactérienne nécessite un examen cytobactériologique des urines (ECBU) et doit être prélevée systématiquement avant toute antibiothérapie. L´ECBU retrouve une leucocyturie ou une pyurie avec une bactériurie [14,24], le seuil de bactériurie dans les prostatites aiguës est fixé à 10 000UFC/ml; l´ECBU étant le seul examen requis [25]; avec antibiogramme avant la mise en route du traitement antibiotique sans oublier qu´un examen normal n´exclut pas le diagnostic [26]. L´imagerie de la prostate est inutile, l´échographie de la prostate ne se justifie que si un abcès prostatique est suspecté (échographie transrectale) ou pour rechercher un résidu vésical (échographie sus pubienne) [27].

Si l´inutilité des hémocultures dans la prise en charge des pyélonéphrites aiguës simples semble établie, leur place dans les prostatites aiguës n´a jamais été étudiée; cependant l´hémoculture est parfois utile apportant quelques fois des diagnostics supplémentaires [28]. Il existe peu de doute quant à l´origine infectieuse des prostatites bactériennes aiguës et même chroniques. Les germes gram négatif sont les plus souvent en cause et la participation des cocci gram positif est discutée [27].

Le traitement repose sur une antibiothérapie probabiliste puis adaptée secondairement et prolongée, un traitement symptomatique efficace, et un traitement spécifique des complications [18]; les antibiotiques sont débutés sans attendre les résultats de l´ECBU et parmi les plus indiqués on retrouve les aminoglycosides, l´azthreoname, les céphalosporines de 3^e^ génération, le cotrimoxazole et surtout les fluoroquinolones et ce, pendant une durée allant de 3 à 6 semaines afin d´éviter les récidives [13]. Une attention particulière devra être faite devant les formes particulières d´*Escherichia coli* résistantes aux fluoroquinolones [29,30].

Le massage prostatique est contre-indiqué et par conséquent l´épreuve des quatre verres de (Meares-Stamey) est inutile [27]. La nécessité d´un traitement bien conduit semble très importante pour éviter les récidives et le passage à la chronicité. La recherche d´une sténose de l´urètre est indispensable [13]. Enfin l´intérêt d´une prise d´un bilan urologique à distance en vue d´une prise en charge secondaire d´une hyperplasie bénigne prostatique sous-jacente est primordial [18] mais aussi hormonal afin de s´assurer qu´il n´existe pas de déficit androgénique chez le patient qui fait une prostatite récidivante ou chronique [31].

## Méthodes

Nous avions réalisé une étude descriptive transversale et rétrospective au service d´urologie du département de chirurgie des Cliniques Universitaires de Lubumbashi. Cette étude menée sur une période de quatre ans soit de Janvier 2015 à Décembre 2018 a concerné les dossiers des patients suivis pour prostatite aiguë non porteur d´une tumeur de la prostate.

Nous nous somme servi d´une fiche d´enquête reprenant différents paramètres d´étude repartis en: les paramètres épidémiologiques: la fréquence, l´âge, la commune de résidence; les paramètres cliniques: les antécédents généraux et urologiques du patient; l´histoire de la maladie qui doit avoir était récente par la survenue brutale des signes cliniques datant de moins d´un mois; les signes cliniques comprenant: syndrome infectieux comprenant essentiellement une hyperthermie (fébricule 37,5 à 38,5 dégrée Celsius et fièvre au-delà de 38,5 dégréé Celsius); syndrome douloureux (douleur hypogastrique, périnéales, lombaire, parfois testiculaire), signes urinaires aussi bien irritatifs (pollakiurie, miction impérieuse) qu´obstructif (dysurie, rétention aiguë d´urine). Le toucher rectal est un élément indispensable pour le diagnostic; les paramètres paracliniques: biologie bilan inflammatoire avec Numération Formule Sanguine (NFS), globules blancs (GB), vitesse de sédimentation (VS) et nous nous sommes basé surtout sur la CRP comme critère majeure de l´inflammation aiguë pour standardiser nos résultats et les comparés aux autres travaux. Le taux normal de CRP dans notre étude varie de 0 à 5 mg/dl. L´hémoculture a aussi parfois était réalisé en cas d´hyperthermie persistante.

L´examen cytobactériologique des urines (ECBU) a été réalisé chez tous les patients et la leucocyturie a été retenue comme critère obligatoire de la prostatite. La culture des urines ou de secrétions prostatiques recueillies par un massage prostatique prudent et minutieux était pratiquée chez quelques patients et l´absence de germe (absence de bactériurie) a conduit au diagnostic de prostatite aiguë non bactérienne par la présence de la leucocyturie au-delà de 5 GB/champ à l´ECBU.

Le diagnostic de prostatite aiguë était posé sur base des éléments cliniques, le toucher rectal et la bactériologie urinaire essentiellement. Les critères d´exclusion étaient: tout dossier de patient ne faisant pas parti de notre période d´étude; tout patient porteur d´une tumeur prostatique aussi bien bénigne que maligne; tout dossier avec absence de certains paramètres essentiels; l´absence de leucocyturie à l´examen cytobactériologique des urines (ECBU); la présence d´un foyer d´infection rénale.

Quarante-deux dossiers de patients avec prostatite aiguë ont été récolté parmi lesquels 17 étaient porteurs d´une tumeur de la prostate dont 13 hypertrophie bénigne et 4 cancers de la prostate. Ainsi nous avions exclus les 17 patients avec prostatites aiguës porteur d´une tumeur prostatique et seulement 25 ont été retenus et ont constitué notre échantillon de base. Ces données ont été encodées dans épi-info version 7. La saisie du manuscrit de l´article a été faite avec Word 2010, les tableaux avec Excel 2010. L´analyse des données a été faite par le calcul des moyennes et son écart-type, des fréquences. La recherche des articles sur internet avait été faite avec le moteur de recherche PubMed.

## Résultats

Durant les 4 années, nous avons colligé 25 cas de prostatite aiguë chez des patients non porteur d´une tumeur prostatique sur un ensemble de 1960 patients admis dans le service de chirurgie, soit une fréquence de 1,27%. En chirurgie urologie, 326 patients admis et suivis, la prostatite aiguë chez des patients non porteurs de tumeur prostatique représente ainsi donc 7,66% des consultations urologiques aux Cliniques Universitaires de Lubumbashi. La tranche d´âge la plus touchée est celle comprise entre 19 - 37 ans avec 64% des cas (Tableau 2); l´âge moyen est de 33,16±2,4 ans; âges extrêmes: 15 et 59 ans.

**Tableau 2:** répartition des cas en fonction de l’âge

Age (ans)	Effectif	Pourcentage
0 – 18	2	8
19 – 37	16	64
38 – 56	6	24
57 – 75	1	4
Total	25	100

La tranche d’âge la plus touchée est celle comprise entre 19 – 37 ans ; âge moyen : 33,16±2,4 ans

En fonction des antécédents, 11 patients sur 25 soit 44% n´avaient aucun antécédent particulier. Parmi les antécédents médicaux, le diabète a été l´antécédent le plus retrouvé avec 4 cas soit 16%. Par rapport aux antécédents urologiques, la dilatation urétrale par usage du béniquet métallique a été le plus retrouvé avec 3 cas soit 12% (Tableau 3). En fonction de la sévérité de l´infection, la majorité n´était pas des formes sévères; 17 patients étaient suivis en ambulatoire soit 62% et 8 seulement en hospitalisation soit 32% pour tableau infectieux sévère (6 cas sur 8) ou association avec une orchi-épidydimite (2 cas sur 8). La durée d´hospitalisation va de 2jours à 15 jours.

**Tableau 3:** répartition des cas en fonction des antécédents

Antécédent	Effectif	Pourcentage
Aucun	11	44
Diabète	4	16
HTA	2	8
HIV	1	4
IST	2	8
Sondage	2	8
Biopsie prostatique	1	4
Prostatite	2	8
Dilatation urétrale par béniquet métallique	3	12

Aucun antécédent n'a été retrouvé chez 11 patients (44%) et la dilatation urétrale est ressortie comme l'antécédent urologique le plus important avec 3cas (12%)

Sur le plan clinique, 3 signes ont dominé la symptomatologie à savoir la fièvre, un syndrome douloureux et une sensibilité de la prostate au toucher rectal avec respectivement 15 cas (60%), 17 cas (68%) et 16 cas (64%) (Tableau 4). En ce qui concerne l´imagerie, l´échographie abdominopelvienne a été le seul examen d´imagerie réalisée, chez 16 patients sur 25, ce qui représente 64% des cas. Par rapport à la biologie, le bilan inflammatoire a été réalisé chez tous nos 25 patients fait de CRP, VS et GB. La CRP étant considéré comme facteur inflammatoire primordial de l´infection aiguë, était élevée chez tous nos 25 patients. L´hémoculture avait été réalisé seulement chez 4 patients (16%) parmi lesquels 3 étaient positives.

**Tableau 4:** répartition des cas en fonction des signes cliniques

Signes cliniques	Effectif	Pourcentage
Fièvre	15	60
Dysurie	8	32
Brulures mictionnelles	11	44
Syndrome douloureux	17	68
Pollakiurie	3	12
Sensibilité de la prostate au toucher rectal	16	64

Prédominance de l'atteinte de la douleur et de la fièvre avec respectivement 68% et 60% des cas

L´ECBU avec antibiogramme, un des critères biologiques majeur était réalisé chez tous nos 25 patients. Dix-neuf cultures étaient positives sur les 25 soit 74% et 6 étaient stériles (24%). En rapport avec les germes, *Escherichia coli* était le germe le plus retrouvé sur les 19 cultures avec 16 cas soit 84,21%, Klebsiella 2 cas soit 10,52% et le Pseudomonas 1 cas soit 5,26% des cas. Sur le plan thérapeutique, tous nos patients ont été soumis à un anti-inflammatoire. Les antibiotiques ont été utilisés chez tous nos patients également. La monothérapie a été utilisée chez 17 patients (68%) tandis que la bithérapie a été administrée chez 8 patients (32%). Concernant l´antibiothérapie, les fluoroquinolones ont été les plus utilisés, chez 16 patients sur les 25 soit 64%. Concernant les 8 autres patients, l´association amoxicilline/acide clavulanique a été utilisée chez 4 patients (16%) (Tableau 5). Concernant l´évolution, 22 patients sur nos 25 ont une évolution favorable soit 88% et 3 ont eu une évolution vers des complications soit 12%.

**Tableau 5:** répartition des cas en fonction des antibiotiques administrés

Antibiotiques	Effectif	Pourcentage
Fluoroquinolone	12	48
Amocicilline/acide clavulanique	2	8
Doxycycline seule	1	4
Imipénème	2	8
Fluoroquinolone + céphalosporine de 3^e^ génération	4	16
Céphalosporine de troisième génération + doxycycline	2	8
Amoxicilline/acide clavulanique + doxycycline	2	8

Les fluoroquinolones sont les antibiotiques les plus utilisés dans le traitement des prostatites aiguës

## Discussion

**Données épidémiologiques:** en rapport avec la fréquence des prostatites, elles sont relativement fréquentes en urologie [4]. Aux USA, sa prévalence est de 5 à 9%; ceci représente près de 2 millions d´hommes traités pour prostatites chaque année [32]. Mais les prostatites bactériennes aiguës et même chroniques sont moins fréquente que les prostatites chroniques abactériennes et les prostatodynies, cependant les études épidémiologiques portant sur les populations citadines manquent [27]. Une étude récente a évalué l´incidence des prostatites bactériennes aiguës à 1,26 cas pour 1000 hommes/an [32]. Les patients avec des signes évidents de prostatite représentaient environ 3% d´hommes canadiens [33] suivis en ambulatoires causant une importante morbidité [34] et coûts [35].

Les prostatites touchent l´homme le plus souvent entre 40 et 60 ans [13], pour certains auteurs elles touchent les jeunes adultes de moins de 50 ans et le troisième diagnostic le plus fréquent chez les hommes de plus de 50 ans [6]. Les études épidémiologiques descriptives montrent que la prostatite affecte les hommes de tout âge [33], ceci a été vu dans notre série ou les âges extrêmes étaient de 17 à 56 ans.

Habituellement, les prostatites aiguës touchent plus volontiers l´homme jeune alors qu´il semble que les prostatites chroniques touchent plus facilement l´homme âgé [13] justifiant ainsi notre observation ou la tranche d´âge la plus touchée est celle de 19 à 37 ans avec 64% des cas pour un âge moyen de 33,16±2,4 ans; notre âge moyen est très inférieure à celui trouvé par Auzanneau *et al*. [21] qui trouve un âge médian de 56,5 ans. Ceci pourrait s´expliquer par le fait que dans notre série, nous avons exclus des patients porteurs d´un adénome de la prostate, ou de cancer ou une hyperplasie de la prostate. Ceux-ci étant plus fréquents chez les patients au-delà de cinquante ans et certaines littératures classifient l´adénomite comme une des trois formes cliniques particulières individualisées de prostatite à côté des formes subaiguës pour lesquelles le tableau d´infection urinaire fébrile est au premier plan et des formes trompeuses, fébriles pures ou douloureuses pures voire un accident de rétention aiguë d´urine [13].

**Données cliniques:** les principaux facteurs de risques de complications sont de prostatites aiguës sont: la prostatite récidivante, un terrain à risque de tumeur de la vessie, des antécédents urologiques, des anomalies morphologiques de l´appareil urinaire et d´autres maladies évolutives responsables d´un déficit immunitaire [18]. Il s´avère impérieux de documenter les antécédents médicaux et chirurgicaux du patient (particulièrement urologiques), la notion de traumatisme, des antécédents toxico-allergiques [36]. Certaines études affirment que la biopsie transrectale est un facteur important favorisant la survenue des prostatites aiguës [28,37] alors que Brede et Shoskes signalent par contre que les patients à haut risque de prostatite aiguë sont spéciale, ayant un antécédent de diabète, de cirrhose ou d´immunosuppression [38]; l´infection étant souvent ascendante [28]. Dans notre étude, une proportion importante des patients n´avaient aucun antécédent particulier (11 patients soit 44%), par contre, un seul patient avec antécédent de biopsie prostatique dans notre étude ne nous permet d´affirmer l´étude de Brede et Shoskes. Cependant, la dilatation urétrale ressort comme antécédent important (12%) chez les patients avec prostatites aiguës sur des prostates non tumorales.

La prostatite aiguë est caractérisée par une infection sévère des voies urinaires, les signes irritatifs et obstructifs avec des signes généraux d´un uro-sepsis [8,28,36]. La fièvre a constitué 73,1% chez Beyung II et la Pyurie (77,9%) étaient les signes les plus fréquents. Antécédent (ATCD) d´alcool (38,9%), tabac (36,3%), diabète (35,1%), indice de masse corporel (IMC) en kg/m^2^ (23,7±4,0), HTA (37,4%), symptômes urinaires (38,6%), douleur (47,9%), rétention (17,4%), manipulations prostatiques antérieures (41,4%), hématurie (54,2%), PSA (ng/ml) (20,2±34,1), volume prostatique (ml) (37,8±17,3) [39].

Dans la série de Lee *et al*. l’âge moyen 63,99±14,27; IMC (kg/m^2^) (24,05±3,79); PSA (ng/ml) (20,76±19,17); volume prostatique (cc) (40,80±17,53); durée des symptômes (jours) (3,04±2,57); antécédents de diabète (48/142), HTA (60/142), pathologies cardiaques (27/134), déficit neurologique(41/142). Insuffisance rénale chronique (20/142), pneumopathie chronique (12/142), cirrhose du foie (22/142), hospitalisations antérieures (24/142), procédés urologiques (13/142), cathétérisme au long court (15/142), cathétérisme récent (13/142), troubles urinaires (30/142). Choc septique (6/142), décès (2/142), évolution vers la chronicité (4/142), durée de traitement (jours) (26,98±9,43) dans le groupe des prostatites aiguës sans abcès prostatiques et beaucoup plus long en cas d´évolution vers un abcès prostatique (40,96±10,61); durée d´hospitalisation (6,69±2,92) dans le groupe de simple prostatite aiguë contre (12,38±6,59) dans le groupe d´abcès prostatiques) [40]. Dans notre étude, la fièvre était également au premier plan avec 60% des cas, puis les signes urinaires comme symptômes majeurs repartis en brulures mictionnelles retrouvées chez 11 patients (44%), la dysurie chez 8 patients (32%), la rétention aiguë d´urine chez 3 patients (12%), un syndrome douloureux chez 17 patients (68%) et surtout un toucher rectal sensible chez 16 patients (64%). Tout ceci corrobore avec les données de la littérature qui affirment que la prostatite aiguë est une infection sévère dominée par des signes urinaires aussi bien irritatifs qu´obstructifs et surtout la fièvre témoignant d´une bactériémie voire une septicémie à porte d´entrée urinaire.

L´examen physique doit examiner l´abdomen, les organes génitaux externes, le périnée, et la prostate. Dans notre étude, nous avons retrouvé 6 cas d´orchi-épidydimite associées à la prostatite (24%). Le massage prostatique pendant l´examen du toucher rectal n´est pas recommandé [6].

**Données paracliniques:** la biologie est un examen indispensable pour étayer le diagnostic de prostatite aiguë. La culture des urines avec l´antibiogramme est d´une importante capitale pour le diagnostic des prostatites aiguës. Celle-ci devrait être réalisé chez tous les patients avec suspicion de prostatite aiguë afin de déterminer le germe responsable et la sensibilité antibiotique y afférente [37]. La cause d´infection prostatique, dans la majorité des cas, bactérienne et les germes les plus couramment rencontrés sont les bactéries gram négatifs, de la famille des entérobactéries dont le chef de fil reste l´*Escherichia coli*, puis Enterobacter, Klebsiella, Serratia, Pseudomonas, et le *Proteus* spp., mais quelques bactéries gram positif, particulièrement Enterococcus, peuvent aussi en être responsables. Les micro-organismes responsables des infections sexuellement transmissibles peuvent également causer une infection prostatique, qui incluent *Neisseria gonorrhoeae, Chlamydia trachomatis, Ureaplasma urealyticum, Mycoplasma hominis, Trichomonas vaginalis*, et *Gardnerella vaginalis* [33,41].

Dans la série de Yoon *et al*. la culture pathogène a isolé comme germes les plus fréquents l´*Escherichia coli* (61,3% groupe A et 61,5% groupe B) suivi de *Pseudomonas* spp. (15,1% groupe 1 et 15,3% groupe B), *Klebsiella* spp. (13,4% groupe A et 9,6% groupe B), E *nterobacter* spp. (7,5% et 7,6%), S *treptococcus agalactiae* (2,7% et 5,7%) [39]. La prédominance d´*Escherichia coli* dans les prostatites aiguës fait l´unanimité dans la quasi-totalité des publications. Ceci est également prouvé par notre étude dans laquelle la fréquence d´*Escherichia coli* est de 16 cultures sur les 19 positives soit 84,21% des cas. Coker *et al*. [37] signale que l´*Escherichia coli* est le germe le plus isolé dans les cultures pouvant aller de 50% à 90% des cultures analysées. Sur les 142 cultures examinées, 21 d´entre elles étaient négatives, ce qui représente un taux de 14,78% et 121 étaient positives soit 85,12%; parmi celles positives, 78 cultures avaient révélées *Escherichia coli*, 14 cultures le *Klebsiella pneumoniae*, et 8 cultures le *Pseudomonas aeruginosa*. Dans notre étude, nous avons constaté que 6 cultures sur 25 étaient stériles, ce qui représente une fréquence de 24% faisant imposer le diagnostic d´une prostatite aiguë abactérienne contre 76% des cultures positives. Sur nos 19 cultures positives, *Escherichia coli* a été retrouvé dans 16 cultures soit 84,21% ce qui corrobore avec les affirmations de Coker *et al*. selon lesquelles l´*Escherichia coli* est le germe le plus isolé dans les cultures pouvant aller de 50% à 90% des cultures analysées.

Malgré que les entérobactéries soient les plus importants germes en cause de prostatite aiguë et chronique, les germes gram positifs et les atypiques sont également quelques fois en cause quoi que leur rôle soient controversés malgré le fait qu´ils soient isolés dans les cultures [40,42]. Malgré la certitude qu´*Escherichia coli* est la cause principale de prostatite aiguë bactérienne, certaines littératures affirment que nous avons encore une connaissance limitée de la virulence associée aux caractéristiques de ce germe dans l´étiopathogénie de la prostatite aiguë bactérienne [26].

La biologie réalisée chez tous nos 25 patients a fait état d´un réel syndrome inflammatoire chez tous nos patients marqués par une accélération de la vitesse de sédimentation et une élévation de la CRP. Cependant, l´hémoculture n´avait été réalisée que chez 4 patients hospitalisés avec un tableau d´urosepsis chez qui 3 cultures étaient positives et une négative. L´échographie transrectale ou le scanner sont indiqués chez des patients avec prostatites réfractaires à la thérapie initiale ou pour exclure un abcès prostatique [6]. L´étude prospective menée par Horcajada *et al*. en 2003 [30] sur 45 patients avait montré que 21 patients sur 45 soit 46,6% des patients avaient des anomalies échographiques prostatiques périphériques.

Malheureusement cet examen n´est pas d´usage courant dans notre pratique expliquant qu´aucun cas d´échographie transrectale n´a été réalisé. Les seules échographies réalisées dans notre étude sont celles abdominopelviennes, réalisées chez 16 patients sur les 25 soit 64%; malheureusement elles ont des limites dans l´appréciation de la prostate par rapport à celles endorectales. Néanmoins dans notre étude, l´échographie abdominopelvienne a permis d´avoir quelques caractéristiques échographiques de la prostate notamment le volume, la surface, et surtout l´exclusion d´une formation d´un abcès prostatique. Les données supplémentaires de laboratoire peuvent être obtenues basées sur les facteurs de risque et la sévérité de la maladie [37]. Le dosage de la PSA n´est pas recommandé [6] justifiant donc le manque de données de PSA dans notre étude. La radiographie n´est pas nécessaire typiquement [37] expliquant l´absence de radiographie à notre étude.

**Données thérapeutiques et évolutives:** les facteurs suivants doivent être pris en compte dans le traitement des prostatites aiguës: le potentiel uro-sepsis, le choix des agents antimicrobiens, le drainage des urines, les facteurs de risques justifiant une hospitalisation et des mesures auxiliaires tendant à améliorer l´issue du traitement [42]. Le choix et la durée du traitement antimicrobien pour la prostatite aiguë est basé sur l´expérience et l´opinion des experts prouvée par des études cliniques [43,44]. Plusieurs études cliniques affirment fortement que la thérapie multimodale est meilleure que la monothérapie chez des patients avec des symptômes à long termes [8,45].

La prostate a une structure unique et des caractéristiques particulières qui rendent certains antibiotiques moins effectifs. Elle tend à être alcaline et ses capillaires ne sont pas aussi perméables que les capillaires d´autres tissus; c´est pourquoi les antibiotiques avec un pKa très élevé et une bonne liposolubilité se retrouvent en grande concentration dans ces tissus. Le fluoroquinolone, les tétracyclines, les macrolides, le trimethoprime (et non la sulfamethoxazole) ont généralement ces caractéristiques. Cependant, l´inflammation aiguë de la prostate permet la pénétration de plusieurs antibiotiques (excepté les nitrofurantoines), qui donnent au clinicien plusieurs options de traitement de la prostatite aiguë [3]. L´antibiothérapie reste la base du traitement pour tous les cas de prostatites aiguës [44] et même chroniques alors que la chirurgie a un rôle limité dans la prostatite aiguë et serait réservée au drainage d´abcès prostatique et à l´ablation des calculs prostatiques infectés [46].

Le traitement initial des patients sévèrement malades, le traitement ci-dessous est recommandé: l´administration intraveineuse des fortes doses d´antimicrobiens bactéricides, tels que la combinaison d´aminoglycosides et ampicilline, une pénicilline associée aux inhibiteurs des beta-lactamases, céphalosporines de troisième génération ou une fluoroquinolone, l´association pipéracilline/tazobactam jusqu´à la défervescence et la normalisation des signes d´uro-sepsis [36,37,47-49]. Les patients n´étant pas sévèrement malades doivent être traités en ambulatoire par une antibiothérapie par voie orale en occurrence une fluoroquinolone [37,43,45].

Dans la série de Yoon *et al*. chez les patients hospitalisés, l´association céphalosporines-aminosides a été la plus administrée avec 58,2% des cas alors que chez les patients ambulants, ce sont les quinolones qui ont été les plus administrés avec 67,9% des cas [50]. Ceci corrobore avec notre observation car dans notre étude, les quinolones ont également été les antibiotiques les plus utilisées avec 64% des cas soit chez 18 patients sur nos 25. Plusieurs études affirment l´efficacité de l´association d´antibiotiques sur la monothérapie [46]. L´association trimethoprime/sulfamethoxazole n´est plus recommandé en première ligne de thérapie empirique dans les milieux ou *E. coli* est résistant à cette association, le plus fréquent agent pathogène, est au-delà de 10 à 20% [6,44,45,51].

Le traitement doit être pris sur deux à quatre semaines [43,45]. C´est ainsi que la durée minimale dans notre observation est de 2 semaines avec comme critère absolue d´arrêt de traitement la stérilisation des urines confirmée par l´ECBU. Trois de nos patients (12%) ont pris les antibiotiques jusqu´à 6 semaines alors que 6 patients (24%) ont eu 4 semaines de traitement et les 16 autres (64%) ont eu un traitement bien conduit pendant 2 semaines.

Le drainage d´urine: le simple cathétérisme avec un cathéter de petit calibre est recommandé chez les patients avec des signes d´une sévère obstruction ou une rétention aiguë d´urine. La pose du cathéter sus-pubien est optimale pour les patients qui ne tolèrent pas le cathéter urétral [8]. Dans la série de Yoon *et al*. la fréquence de cystostomie était de 49,4% dans le groupe A contre 73,1% dans le groupe B; le cathétérisme urétral de 9,6% contre 53,8% [43]. La déviation des urines par cystocathéther est également réalisée en cas de rétention aiguë d´urine comme c´était le cas des 3 de nos patients (12%); cependant, aucun cas de cystotomie n´a été réalisé dans notre série.

L´hospitalisation est recommandée en cas d´hyperpyrexie, vomissements prolongés, déshydratation sévère, tachycardie, tachypnée, hypotension et autres symptômes traduisant un uro-sepsis. Elle est recommandée chez des patients à haut risque (diabète, patients immunodéprimés, patients âgés ou porteur d´un abcès prostatique) et ceux avec un trouble mictionnel sévère [6]. Dans la série de Lee *et al*. sur 142 patients, 101 étaient admis en hospitalisation alors que 41 étaient suivis en ambulatoires [40], ce qui représente environ 71,12% d´hospitalisation dans sa série. Ce taux est grandement plus élevé que le nôtre qui est de 32% seulement d´hospitalisation. Nous pensons donc que, notre fréquence faible d´hospitalisation est expliquée par la forte automédication des patients avant de consulter, ce qui masque certains symptômes tels que la fièvre et les signes urinaires qui, normalement sont les indications d´hospitalisation.

Mesures auxiliaires: les anti-inflammatoires non stéroïdiens ont été suggéré pour réduire les symptômes incluant la fièvre [36,43]. Les alpha-bloquants devaient être considérés, particulièrement chez les patients avec des signes modérés d´obstruction des voies urinaires dans le but de réduire le risque de rétention urinaire et faciliter la miction [33,43,45]. Ce qui corrobore avec notre observation où tous nos patients sont soumis ipso facto aux anti-inflammatoires non stéroïdiens (AINS) dès la pose du diagnostic. Cependant, l´usage des α-bloquants n´est pas de coutume dans notre maison de formation, expliquant qu´aucune prescription de ces médicaments ne soit faite.

Malgré le traitement, les patients ayant fait une prostatite aiguë ont un risque de 20 à 50% de développer une récidive [24] et environ 5% des patients avec prostatite aiguë bactérienne, l´inflammation peut devenir chronique [36,47] voire 10% [15]. Dans notre série, 3 patients sur 25 (12%) ont évolué vers une prostatite chronique alors que dans la série de Yoon *et al*. seulement 6 patients sur 437 avec prostatites aiguës (1,3%) ont évolué vers une prostatite chronique [50]. Notre faible échantillon expliquerait certainement cette différence en pourcentage. Certaines études rapportent que les patients répondant mieux aux antibiotiques ont une bonne issue par contre ceux ne répondant pas aux traitements ou ceux qui ne sont pas traités devraient avoir un faible taux de fertilité. Certaines autres études rapportent que les patients avec une importante charge bactérienne ont une tendance à avoir une diminution de mobilité des spermatozoïdes et de leur viabilité [26,40]. Malheureusement notre étude n´a pas été conduite dans cette optique, et il faudrait une étude de suivi prospective et approfondie pour affirmer cette théorie.

L´abcès prostatique est une complication également fréquente en cas de prostatite aiguë même si dans notre série nous n´avons pas eu de cas ayant évolué vers cette complication. Carroll *et al*. signale que l´abcès prostatique est une complication rare de la prostatite bactérienne aiguë et est fréquemment causée par une entérobactérie quoique le *Staphylococcus aureus* soit également décrit dans une publication [42]. Cependant, malgré que les complications soient rares de nos jours en cas de prostatite aiguë, une étiologie particulière et un groupe particulier tel que chez les patients infectés par le HIV, existent des difficultés énormes de diagnostic et de traitement chez qui apparait une forte restauration rapide de la fonction immunitaire par le traitement antirétroviral qui est donc plus qu´important chez eux [44].

Dans la série de Lee *et al*. 31 patients sur 142 avec prostatite aiguë ont développé un abcès prostatique. L´analyse uni-variée a révélé que la durée des symptômes, le diabète et les troubles urinaires étaient des facteurs prédisposant à la formation des abcès sur prostatites aiguës [50]. Cependant, Millán-Rodríguez affirment que les antécédents de manipulations des voies urinaires constituent un haut risque d´évolution vers un abcès prostatique et des multiples infections aux germes autres que *Escherichia coli* [52] bien qu´un cas d´abcès prostatique soit décrit dans la littérature [53].

Le diagnostic précoce est utile car l´abcès prostatique requiert une prolongation de traitement associée à un drainage chirurgical de l´abcès [40].

## Conclusion

La prostatite aiguë sur prostate non tumorale reste une entité nosologique urologique très préoccupante dont la prise en charge doit être rigoureuse d´autant plus que la population à risque est celle en période d´intense activité sexuelle. L´usage de l´échographie endorectale, la proscription du massage prostatique doivent s´intégrer dans la prise en charge aux cliniques universitaires de Lubumbashi.

### Etat des connaissances sur le sujet

- Les prostatites aiguës sont très fréquentes en urologie;
- Escherichia coli est le germe le plus incriminé;
- L´abcès prostatique est une complication redoutable et assez fréquente.

### Contribution de notre étude à la connaissance

- La fréquence élevée des prostatites aiguës chez des patients jeunes non porteurs d´une tumeur prostatique à Lubumbashi;
- Le profil épidémio-clinique et thérapeutique des prostatites aiguës chez des patients non porteurs de tumeurs prostatiques aux cliniques universitaires de Lubumbashi;
- Les facteurs favorisants des prostatites aiguës en dehors des tumeurs prostatiques chez les jeunes sont dominés par la dilatation urétrale.

## Remerciements

Nos remerciements s´adressent aux médecins stagiaires qui nous ont aidé dans la récolte des données.
